# Performance of algorithms for tuberculosis active case finding in underserved high-prevalence settings in Cambodia: a cross-sectional study

**DOI:** 10.1080/16549716.2019.1646024

**Published:** 2019-09-05

**Authors:** Kimcheng Choun, Tom Decroo, Tan Eang Mao, Natalie Lorent, Lisanne Gerstel, Jacob Creswell, Andrew J. Codlin, Lutgarde Lynen, Sopheak Thai

**Affiliations:** a Infectious Disease Department, Sihanouk Hospital Center of HOPE, Phnom Penh, Cambodia; b Department of Clinical Sciences, Institute of Tropical Medicine, Antwerp, Belgium; c Research Foundation Flanders, Brussels, Belgium; d National Center for Tuberculosis and Leprosy Control, Phnom Penh, Cambodia; e Respiratory Diseases Department, University Hospitals Leuven, Leuven, Belgium; f KIT Royal Tropical Institute, KIT Health, Amsterdam, The Netherlands; g Stop TB Partnership, Geneva, Switzerland

**Keywords:** Outreach, chest X-ray, Xpert MTB/RIF, sputum smear microscopy, clinical diagnosis

## Abstract

**Background**: Most studies evaluate active case findings (ACF) for bacteriologically confirmed TB. Adapted diagnostic approaches are needed to identify cases with lower bacillary loads.

**Objectives**: To assess the likelihood of diagnosing all forms of TB, including clinically diagnosed pulmonary and extra-pulmonary TB, using different ACF algorithms in Cambodia.

**Methods**: Clients were stratified into ‘high-risk’ (presumptive TB plus TB contact, or history of TB, or presumptive HIV infection; n = 12,337) and ‘moderate-risk’ groups (presumptive TB; n = 28,804). Sputum samples were examined by sputum smear microscopy (SSM) or Xpert MTB/RIF (Xpert). Initially, chest X-ray using a mobile radiography unit was a follow-up test after a negative sputum examination [algorithms A (Xpert/X-ray) and B (SSM/X-ray)]. Subsequently, all clients received an X-ray [algorithms C (X-ray+Xpert) and D (Xray+SSM/Xpert)]. X-rays were interpreted on the spot.

**Results**: Between 25 August 2014 and 31 March 2016, 2217 (5.4%) cases with all forms of TB cases were diagnosed among 41,141 adults. The majority of TB cases (1488; 67.1%) were diagnosed using X-ray. When X-rays were taken and interpreted the same day the sputum was collected, same-day diagnosis more than doubled. Overall, the number needed to test (NNT) to diagnose one case was 18.6 (95%CI:17.9–19.2). In the high-risk group the NNT was lower [algorithm D: NNT = 17.3(15.9–18.9)] compared with the ‘moderate-risk group’ [algorithm D: NNT = 20.8(19.6–22.2)]. In the high-risk group the NNT was lower when using Xpert as an initial test [algorithm A: NNT = 12.2(10.8–13.9) or algorithm C: NNT = 11.2(9.6–13.0)] compared with Xpert as a follow-up test [algorithm D: NNT = 17.3(15.9–18.9)].

**Conclusion**: To diagnose all TB forms, X-ray should be part of the diagnostic algorithm. The combination of X-ray and Xpert testing for high-risk clients was the most effective ACF approach in this setting.

## Background

Tuberculosis (TB) remains the leading cause of death from an infectious disease, causing 1.6 million deaths in 2017 [1]. Although improved access to and quality of TB care have reduced TB-related mortality since 1990, more than one third of an estimated 10 million new cases of active TB remain undiagnosed every year, which is a major reason for the slow decline in TB incidence [1].

Passive case finding alone, waiting for people to seek health care, will not suffice to stop transmission of TB in high prevalence settings [1,2]. Active case finding (ACF), the screening and testing for TB among people not spontaneously presenting to health facilities, aims to reach more people, diagnose and treat TB earlier, and reduce the period of infectiousness [2]. In settings with a high prevalence of undiagnosed TB, the World Health Organization (WHO) recommends ACF for high risk groups, such as TB contacts and people living with HIV, and populations with poor access to health care, such as people living in remote areas or urban slums [2,3].

ACF encompasses many different approaches and it is currently unknown what strategies are most effective in different screening settings. Ideally, a diagnostic algorithm consists of one or more tests which (1) can be provided in the community, (2) have a short turn-around time, (3) are accurate, with a high sensitivity and specificity, and (4) assist in identifying all forms of TB, including bacteriologically confirmed pulmonary TB (BC PTB), clinically diagnosed PTB (CLIN PTB) and extra-pulmonary TB (EPTB) [4]. Currently, tests used in algorithms do not combine these four characteristics [5]. Hence, flexibility is needed when designing ACF approaches [6].

Many ACF studies have focused on community outreach and introducing new diagnostics to improve case finding. Often community health workers were involved in sputum collection and transport, which led to a focus on bacteriologically confirmed cases [5–10]. However, ACF interventions identify people earlier in their disease history and possibly with lower bacillary loads and a negative sputum examination [2,5]. ACF algorithms increasingly include Xpert MTB/RIF testing [11–13], which has a higher sensitivity than sputum smear microscopy (SSM) [14]. However, if the number needed to test (NNT) with Xpert MTB/RIF to diagnose one case with BC PTB is high, this approach is costly and CLIN PTB is overlooked [5]. Chest X-ray (CXR) screening followed by Xpert MTB/RIF testing has been proposed to reduce the NNT to diagnose once case with BC PTB [5]. Moreover, adding CXR to the ACF algorithm provides an opportunity for the diagnosis of CLIN PTB.

Few studies evaluated ACF for the diagnosis of all forms of TB [15–17]. One Cambodian study studied mobile CXR followed by SSM for active and passive case finding and concluded that ACF was cost-effective [15]. Another Cambodian study targeting elderly showed that an algorithm using Xpert and CXR increased TB diagnosis [16]. Two-thirds were clinically diagnosed, and such case finding was enhanced by the use of CXR [16]. However these studies did not compare different algorithms. A study from China used survey-data to model the yield of different ACF algorithms for the diagnosis of all forms of TB in elderly people (65 years or older) and recommended the use of CXR as an initial test [17].

Between 2014–2016, a Cambodian ACF program aimed at diagnosing all forms of TB in individuals with presumptive TB (cough, fever, night sweats or weight loss for a minimum duration of 2 weeks [18]). Different diagnostic algorithms were used. All algorithms included symptom screening, CXR and sputum examination. To add to the limited body of evidence on ACF for all forms of TB, in this paper we aim to assess the likelihood of diagnosing all forms of TB, including CLIN PTB and EPTB, combining CXR and sputum examination in different diagnostic algorithms during ACF activities in Cambodia.

## Methods

### Study design

Cross-sectional study using program data collected during TB ACF activities in Phnom Penh and Kandal province, in Cambodia.

### Setting

Cambodia is one of the 30 high TB burden countries [1]. In Cambodia, the incidence of all forms of TB is estimated to be 326/100,000 [1]. In 2014 72% case detection treatment coverage was achieved. The TB prevalence reduced with more than 50% between 1990 and 2015 [19]. In 2014, up to 73% of notified cases were referred by community health workers [19]. Of 43,738 TB cases notified in 2014, only 12,613 (29%) were bacteriologically confirmed [19].

The Cambodian TB program offers diagnosis and treatment services for TB patients free-of-charge across the country. The country counted 861 health centers (HCs) in 2015. Primary health care services include sputum collection, smear preparation and TB treatment. SSM reading is done in district hospitals. Provincial referral hospitals provide CXR.

### Intervention area

Phnom Penh, the capital of Cambodia, and the surrounding province of Kandal, have an estimated population of 1,773,000 and 1,423,000, respectively [20]. Between 25 August 2014 and 31 March 2016 catchment areas of HCs with case detection rates lower than the national average were prioritized for ACF. Hence, ACF was organized in the catchment areas of 25 of 34 HC in Phnom Penh and 63 of 101 HC in Kandal province. The Sihanouk Hospital Center of HOPE, an hospital providing free medical care in Phnom Penh and involved in TB care since 1999, coordinated the ACF activities.

### Participants

All adults (15 years or older) with presumptive TB who attended ACF procedures. Between 25 August 2014 and 31 March 2016 were included in study. They were defined in two groups. A ‘high-risk group’ included persons with presumptive TB plus an additional ‘high-risk factor’ – either a history of TB, being a household contact of a TB patient, or presumptive HIV infection (identification of opportunistic infections or self-reported previous HIV diagnosis). A second ‘moderate-risk group’, which included persons with presumptive TB but who did not have any additional ‘high-risk factor’.

### ACF procedures

People identified by village health volunteers with presumptive TB were invited to a central venue within the community, where ACF program staff collected a sputum sample and/or performed a CXR during a clinical consultation. Box 1 shows ACF preparation, procedures, and coordination.

**Box 1.** ACF preparation, procedures, and coordination.

**ACF preparation and training**
● The TB staff from the HCs were invited for a refresher training on smear preparation and treatment registration.
● Laboratory staff working in district hospitals were trained on the smear reading using LED microscopy.
● VHVs and the village leaders were invited for a training organized in the local HC. During the training the purpose of the ACF, as well as the ACF procedures were explained to the local VHVs and the village leaders. After the training the VHVs and the village leaders mapped the number and size of households in the community. They informed the community members about ACF, the planned arrival date of the MXU, and the venue where the MXU would be operating. People with TB symptoms were advised to come for CXR screening.
**ACF procedures**
● The MXU moved from village to village and stationed at central venues like schools or community center, used regularly for various public activities. In each community the MXU stayed for a few days.
● On the day the mobile team arrived in the community, a door-to-door TB symptom screening was done by the VHVs in collaboration with the TB officers. All individuals with presumptive TB were referred to the MXU for sputum collection and CXR.
● Two sputum samples were collected with an interval of half an hour and stored in a cool box. Clients unable to expectorate sputum were eligible for CXR. Moreover, when the SSM and/or Xpert came back negative, clients were traced to have a CXR as second diagnostic test. From 16/03/2015 onwards procedures changed. A CXR was taken the same day sputum samples were collected, and interpreted on the spot by the MXU physician, during a clinical consultation. Hence, the CXR result was available on the spot, in the community, and earlier than the SSM result.
**Flow of samples and feedback-loop**
● As different algorithms applied for different target groups, sputum samples were separated in different cool boxes. A cool box with sputum samples for smear microscopy was sent to the local HC where smears were prepared using glass slides. The same day the slides were sent for LED SSM reading at the district hospital’s laboratory. A cool box with sputum samples for Xpert testing was sent to the SHCH or CENAT laboratory.
● An external quality control of results from LED SSM and X-ray reading was conducted monthly.
● Results of the SSM or Xpert were reported by the laboratory staff to the ACF project coordinator and the TB officer using a short message system (SMS).
● When clients were diagnosed with TB, and not yet put on treatment by the MXU team, the TB officer contacted the VHVs or Village leaders to find the patient and refer them to the local HC for TB treatment initiation.
● A standardized first line TB treatment regimen was used at the local HC. Those who were diagnosed with rifampicin-resistant TB were referred to the CENAT to start the MDR TB treatment regimen.
**Coordination**
● TB officers met every two weeks to report problems and discuss solutions with the ACF project coordinator, who was responsible for the MXU staff, the preparation of ACF in the HC, and the tracking of patients lost to follow-up.
● The ACF Grant Director, the ACF Project Coordinator, met those responsible for monitoring and evaluation and laboratory testing each week to share updates and identify solutions for reported problems.

ACF: active case finding; CENAT: National Center for Tuberculosis and Leprosy Control; CXR: chest X-ray; HC: health center; MXU: Mobil X-ray unit; SHCH: Sihanouk Hospital Center of HOPE; SSM: sputum smear microscopy; TB: tuberculosis; VHV: Village Health Volunteer

Three mobile X-ray units (MXU) visited communities during the intervention. Each MXU consisted of one physician, trained in X-ray reading, two X-ray technicians, one TB nurse, and up to six TB officers trained in TB diagnosis and data collection. The MXU were equipped with a portable X-ray machine. CXRs were performed and interpreted on-site. The hospital laboratory tested collected sputum samples, using Light Emitting Diode (LED)-based fluorescence microscopy (iLED Primostar, Zeiss, Germany), and Xpert MTB/RIF assay (Cepheid SAS, Sunnyvale, USA) (Xpert). Diagnostic algorithms differed for the two target groups (Table 1).

**Table 1.** Algorithms used for active case finding in Cambodia, by target group, between 25 August 2014 and 31 March 2016.

Target group	Period	Algorithm^b^	Sequence of CXR and sputum sampling and testing in patients with TB symptoms^c^
Presumptive TB with additional risk factors: ‘high-risk group’^a^	25/08/2014–15/03/2015	A Xpert/CXR	Xpert testing was the first diagnostic test. Those with a negative Xpert and for those who could not provide sputum, a second visit was done to conduct a CXR. This subgroup has a higher risk of developing TB and an increased risk of resistance to rifampicin.
	16/03/2015 − 14/07/2015	C CXR+Xpert	CXR and sputum collection were done on the same day to reduce the follow-up visits and to initiate more clients on the same day that they were tested in the community.
	15/07/2015–31/03/2016	D CXR+SSM/Xpert	In patients with TB symptoms CXR and sputum collection were done on the same day. When the CXR was abnormal or SSM was negative Xpert testing was done.
Presumptive TB without additional risk factors: ‘moderate-risk group’	25/08/2014–15/03/2015	B SSM/CXR	SSM was the first test. For those with a negative smear and for those who could not provide sputum, a second visit was made to conduct a CXR. To control costs this algorithm used SSM instead of Xpert.
	16/03/2015 and 31/03/2016	D CXR+SSM/Xpert	In patients with TB symptoms CXR and sputum collection were done on the same day. When the CXR was abnormal or SSM was negative Xpert testing was done.

CXR: chest X-ray; SSM: sputum smear microscopy; TB: tuberculosis; Xpert: Xpert MTB/RIF assay, HC: health center; MXU: Mobil X-ray unit

^a^Clients with presumptive TB were allocated in the ‘high-risk group’ if they had an additional ‘high-risk factor’, which included presumptive HIV infection (identification of opportunistic infections or self-reported previous HIV diagnosis), history of TB, and household contact with a TB patient. Clients with presumptive TB but without an additional risk-factor were grouped in the ‘moderate-risk group’.

^b^TB diagnosis was based on bacteriological confirmation (SSM or Xpert) or clinical signs (physical examination, CXR)

^c^Under algorithm C and D, clients with TB signs on the CXR received a first dose of treatment from the MXU physician and were referred to the local HC to continue TB treatment. In such patients the sputum testing served to classify TB in BC PTB and CLIN PTB.

### TB diagnosis

A clinician made the diagnosis of TB based on a positive SSM or Xpert result, and/or a CXR findings compatible with TB, and/or clinical examination showing signs of TB.

Different diagnostic algorithms combining CXR and sputum testing were used in a consecutive manner and in different target groups. Initially, CXR was used as a follow-up test after a negative sputum examination. Subsequently, all clients received a CXR, which was interpreted on the spot. The characteristics of the different algorithms are summarized in Table 1.

BC PTB was defined as a patient with at least one positive smear or one positive Xpert test. A case of CLIN PTB had to have two negative smears or one negative Xpert test, and/or have a CXR with signs suggestive for TB reported by a trained TB physician or radiologist. EP TB was usually diagnosed based on clinical grounds. A diagnosis of TB adenitis (EPTB) was based on symptoms and signs, including presence of a cervical or axillary lymph node bigger than 2 cm and general asthenia (defined as less active in routine activities).

### Variables and data collection

ACF data and laboratory results were encoded in a Microsoft Access database. The data management team communicated back and forth with the TB officers and the laboratory staff to complete missing data or to clean errors or inconsistent information. Collected variables included: age, sex, history of TB, household contact with a TB patients, presumptive HIV infection, self-report of diabetes, self-report of chronic obstructive pulmonary disease, presence of malnutrition, TB symptoms (cough, fever, night sweats, weight loss), the duration of TB symptoms in days, presence of shortness of breath, presence of lymph nodes (cervical or axillary), CXR result, SSM result, Xpert result, date of testing, date of TB treatment initiation.

### Statistical methods

Proportions were calculated for categorical variables and median and interquartile ranges (IQR) for numeric variables. 95% confidence intervals were calculated for proportions. The NNT to diagnose one case of TB was calculated as the number of individuals tested divided by the number diagnosed with TB.

To estimate predictors of TB diagnosis, a multivariate logistic regression model was conducted separately for the ‘high-risk’ and the ‘moderate-risk group’. Adjusted odds ratios (aOR), with 95% confidence intervals (CI), were calculated. P-values of 0.05 or lower were considered as significant. Analyses were performed using Stata (version 14.1, College Station, Texas).

The study was approved by the Institutional Review Board of the Institute of Tropical Medicine, Antwerp.

## Results

**Figure 1.**
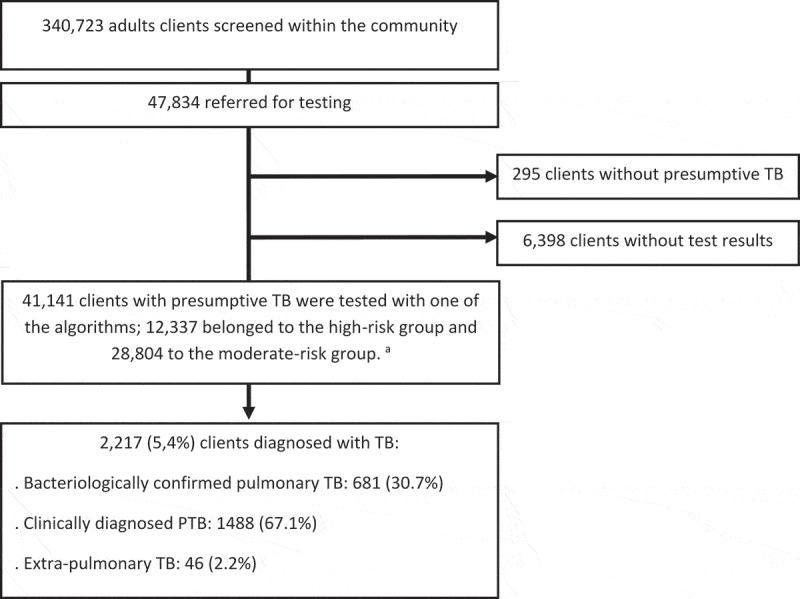
Flowchart showing uptake of diagnostic algorithms during TB active case finding, between 25 August 2014 and 31 March 2016, in Cambodia.

Between 25 August 2014 and 31 March 2016, 340,723 adults were verbally screened for TB symptoms. 41,141 had presumptive TB and had results for one of the diagnostic algorithms (Figure 1). Table 2 shows the characteristics of the adults with presumptive TB; 64.7% were 50 years or older and 37.4% were male. 12,337 belonged to the high-risk group and 28,804 belonged to the moderate-risk group. The most frequently reported TB symptoms included self-reported fever for more than two weeks (present in 85.7%), shortness of breath (84.0%), cough for two weeks or more (80.9%), and night sweats for two weeks or more (57.1%).

**Table 2.** Characteristics of clients tested for TB after a positive symptom screen in the community, between 25 August 2014 and 31 March 2016, in Cambodia.

	High-risk group^a^		Moderate-risk group		Total	
	N	%	N	%	N	%
Total tested	12,337	100.0	28,804	100.0	41,141	100.0
Male	4645	37.7	10,725	37.2	15,370	37.4
Age group:						
15–24	615	5.0	1336	4.6	1951	4.7
25–49	3876	31.4	8705	30.2	12,581	30.6
≥50	7846	63.6	18,763	65.1	26,609	64.7
Symptoms or signs:						
2 weeks fever	10,751	87.1	24,515	85.1	35,266	85.7
Shortness of breath	10,364	84.0	24,194	84.0	34,558	84.0
2 weeks cough	10,085	81.7	23,212	80.6	33,297	80.9
2 weeks night sweats	7382	59.8	16,090	55.9	23,472	57.1
2 weeks weight loss	3419	27.7	5791	20.1	9210	22.4
Lymph nodes	180	1.5	229	0.8	409	1.0
Presumptive HV	125	1.0	0	0.0	125	0.3
TB contact	8341	67.6	0	0.0	8341	20.3
TB history	5465	44.3	0	0.0	5465	13.3
Diabetes Mellitus	258	2.1	663	2.3	921	2.2
Malnutrition	2996	24.3	5213	18.1	8209	20.0
COPD	1033	8.4	861	3.0	1894	4.6
Algorithms: A: Xpert/CXR	2812	22.8	0	0.0	2812	6.8
B: SSM/CXR	0	0.0	4561	15.8	4561	11.1
C: CXR+Xpert	1824	14.8	0	0.0	1824	4.4
D: CXR+SSM/Xpert	7701	62.4	24,243	84.2	31,944	77.6

N: number; CXR: chest X-ray; SSM: sputum smear microscopy; TB: tuberculosis; COPD: chronic obstructive pulmonary disease

^a^Clients with presumptive TB were allocated in the ‘high-risk group’ if they had an additional ‘high-risk factor’, which included presumptive HIV infection (identification of opportunistic infections or self-reported previous HIV diagnosis), history of TB, and household contact with a TB patient. Clients with presumptive TB but without an additional risk-factor were grouped in the ‘moderate-risk group’.

TB: tuberculosis.

^a^ Clients with presumptive TB were allocated in the ‘high-risk group’ if they had an additional ‘high-risk factor’, which included presumptive HIV infection (identification of opportunistic infections or self-reported previous HIV diagnosis), history of TB, and household contact with a TB patient. Clients with presumptive TB but without an additional risk-factor were grouped in the ‘moderate-risk group’.

Among 41,141 adults with presumptive TB, 2,217 (5,4%) were diagnosed with TB: 681 (30.7%) with BC PTB, 1488 (67.1%) with CLIN PTB, and 46 (2.2%) with EPTB (Table 3). In the ‘high-risk group’, the NNT was lower when Xpert was used as first test (algorithm A: NNT = 12.2;95%CI:10.8–13.9) or as first test in combination with CXR (algorithm C: NNT = 11.2;95%CI:9.6–13.0) than when Xpert was used as follow-up test after CXR and SSM (algorithm D: NNT = 17.3;95%CI:15.9–18.9) (both p-values < 0.001). When the same algorithm (algorithm D) was used in both the ‘high-risk’ and the ‘moderate-risk group’, the NNT was lower in the ‘high-risk group’ (17.3 [95% CI:15.9–18.9] versus 20.8 [95%CI:19.6–22.2]) (p-value < 0.001).

**Table 3.** Number needed to test (after a positive symptom screen) to diagnose one TB case, by algorithm, between 25 August 2014 and 31 March 2016, in Cambodia.

				Among those diagnosed: type of TB							
	Total tested	Diagnosed with TB		BC PTB		CLIN PTB		EPTB			
	N	N	(%)	N	(%)	N	(%)	N	(%)	NNT	95%CI
**High-risk group**^a^											
A: Xpert/CXR	2812	230	8.2	72	31.3	144	62.6	14	6.1	12.2	(10.8–13.9)
C: CXR+Xpert	1824	163	8.9	34	20.9	129	79.1	0	0.0	11.2	(9.6–13.0)
D: CXR+SSM/Xpert	7701	445	5.8	148	33.3	293	65.8	4	0.9	17.3	(15.9–18.9)
**Moderate-risk group**											
B: SSM/CXR	4561	213	4.7	60	28.2	138	64.8	15	7.0	21.4	(18.9–24.4)
D: CXR+SSM/Xpert	24,243	846	4.8	367	31.5	784	67.2	13	1.3	20.8	(19.6–22.2)
Total	41,141	2217	5.4	681	30.7	1488	67.1	46	2.2	18.6	(17.9–19.2)

BC PTB: bacteriologically confirmed pulmonary TB; CLIN PTB: clinically diagnosed PTB; EPTB extra-pulmonary TB; Xpert: Xpert MTB/RIF assay; CXR: chest X-ray; SSM: sputum-smear microscopy; N: number; TB: tuberculosis; NNT: number needed to test using an algorithm to diagnose one case; CI: confidence interval

A forward slash ‘/’ shows that test are done sequentially, whereby the next test is done in those who test negative on the previous test. A plus sign “+“ shows that tests are done simultaneously.

^a^Clients with presumptive TB were allocated in the ‘high-risk group’ if they had an additional ‘high-risk factor’, which included presumptive HIV infection (identification of opportunistic infections or self-reported previous HIV diagnosis), history of TB, and household contact with a TB patient. Clients with presumptive TB but without an additional risk-factor were grouped in the ‘moderate-risk group’.

The probability of TB diagnosis was higher among men in both the high-risk group (aOR 1.4; 95% CI:1.2,1.6; p < 0.001) and the moderate-risk group (aOR 1.7; 95% CI:1.6,2.0; p < 0.001). Compared to persons between 25 and 49 years old, the probability of TB diagnosis was higher among persons older than 50 years in both the high-risk group (aOR 1.6; 95% CI:1.4,1.9; p < 0.001) and the moderate- risk group (aOR 2.3; 95% CI:2.0,2.6; p < 0.001) (Table 4).

**Table 4.** Predictors of TB diagnosis, during active case finding in the community, between 25 August 2014 and 31 March 2016, in Cambodia.

	High-risk^a^		Moderate-risk^a^	
	aOR	[95% CI]	aOR^§^	[95% CI]
Sex				
Female	1		1	
Male	1.4***	[1.2,1.6]	1.7***	[1.6,2.0]
Age groups				
15–24	0.8	[0.5,1.2]	0.7	[0.5,1.1]
25–49	1		1	
≥50	1.6***	[1.4,1.9]	2.3***	[2.0,2.6]
Algorithms: A: Xpert/CXR	1.4***	[1.2,1.7]		
B: SSM/CXR			0.9	[0.8,1.1]
C: CXR+Xpert	1.6***	[1.3,1.9]		
D: CXR+SSM/Xpert	1		1	

^a^OR: adjusted odds ratio; CXR: chest X-ray; CI: confidence interval

* p < 0.05 **p < 0.01 *** p < 0.001

**^§^**Adjusted for potential confounders, including gender, age, and TB symptoms (aOR for each of the TB symptoms not shown in the table)

^a^Clients with presumptive TB were allocated in the ‘high-risk group’ if they had an additional ‘high-risk factor’, which included presumptive HIV infection (identification of opportunistic infections or self-reported previous HIV diagnosis), history of TB, and household contact with a TB patient. Clients with presumptive TB but without an additional risk-factor were grouped in the ‘moderate-risk group’.

In the ‘high-risk group’ diagnosis of TB was more likely when Xpert was used as the first test (algorithm A; aOR 1.4 [95%CI: 1.2–1.7]) or as first test in combination with CXR (algorithm C; aOR 1.6 [95%CI: 1.3–1.9])

Overall, 46 (2.1%) TB cases were pre-treatment lost to follow up (Table 5). The median time from test to treatment was 6 days (IQR: 0–14 days) and did not significantly differ between the different algorithms. The proportion of patients who started the same day they underwent testing was higher when a CXR was done and interpreted the same day the sputum samples were collected (as in algorithms C and D).

**Table 5.** Same-day treatment initiation and diagnostic lost to follow-up during active case finding in the community, between 25 August 2014 and 31 March 2016, in Cambodia.

	Total diagnosed	Same-day initiation		Diagnostic LTFU	
	N	N	% (95% CI)	N	% [95%CI]
**High-risk group**^a^					
A: Xpert/CXR	230	10	4.3 (2.1–7.9)	10	4.3 [2.1–7.9]
C: CXR+Xpert	163	37	22.7 (16.5–29.9)	0	0.0 [0.0–2.2]
D: CXR+SSM/Xpert	445	144	32,4 (28.0–36.9)	8	1.8 [0.8–3.5]
**Moderate-risk group**					
B: SSM/CXR	213	28	13.1 (8.9–18.4)	8	3.8 [1.6–7.3]
D: CXR+SSM/Xpert	1166	656	42.0 (39.2–44.9)	20	1.7 [1.1–2.6]
Total	2217	711	31.9 (30.0–33.9)	46	2.1 [1.5–2.8]

N: number; CXR: chest X-ray; SSM: sputum smear microscopy; TB: tuberculosis; LTFU: Loss to follow-up; CI: confidence interval

^a^Clients with presumptive TB were allocated in the ‘high-risk group’ if they had an additional ‘high-risk factor’, which included presumptive HIV infection (identification of opportunistic infections or self-reported previous HIV diagnosis), history of TB, and household contact with a TB patient. Clients with presumptive TB but without an additional risk-factor were grouped in the ‘moderate-risk group’.

## Discussion

ACF interventions using CXR with clinical reading by radiologists in combination with Xpert can identify large numbers of people with TB who may not be reached through passive approaches. As prevalence surveys have found, a large proportion of people with TB need CXR in order to be diagnosed [21].

People diagnosed with TB through ACF often have paucibacillary TB [21]. Studies have also shown low sensitivity of Xpert in ACF scenarios, which emphasizes the importance of TB diagnosis bases on clinical signs [22]. Another Cambodian study showed 71.4% CLIN PTB in patients diagnosed through ACF, compared to 40.5% CLIN PTB in patients diagnosed through passive case finding [15]. In contrast with previous ACF approaches in Cambodia, where 94.1% of all TB diagnoses were bacteriologically confirmed [7], this study aimed to diagnose all forms of TB in this intervention. Hence, many diagnoses were based on CXR and only 30.7% had BC PTB. The lower proportion of BC PTB is consistent with national reporting [19].

In the ‘high-risk group’ the algorithm that provided same-day CXR and sputum collection for Xpert testing was more efficient: it had a lower NNT than the algorithm using Xpert as follow-up test after CXR and SSM and a higher proportion of same-day treatment initiation compared to the algorithm that used CXR as follow-up test after Xpert. Our findings align with findings from a South African study on ACF in TB contacts, which showed 0.5% and 1.6% positivity for SSM and Xpert testing, respectively [23]. When the same algorithm was used, the NNT was lower in the ‘high-risk-group’ compared to the ‘moderate-risk group’. This finding supports the WHO’s recommendations, to target high-risk groups [2].

Treatment delay is increased in algorithms that require two consecutive visits as it is sometimes challenging to track individuals for follow-up testing [4]. Also, in the ‘moderate-risk group’ when CXR and sputum collection for SSM were done together, treatment was more likely to start that same day.

Individuals being 50 years or older were more likely to be diagnosed with TB compared with persons aged between 25 and 49 years. This finding is consistent with the national TB prevalence survey findings in Cambodia, which showed that 55% of BC PTB was notified among the people above 50 years of age [24]. Considering the important role of ACF for diagnosing TB in this subgroup [17,24,25], and the extensive contact between older individuals and household members [26], it seems justified to continue prioritizing this subgroup in future ACF programs. A recent Cambodian study showed that ACF can substantially increase TB diagnosis and treatment among this population [16].

Our study showed that mobile CXR for ACF is feasible and effective. Overall, the delay between diagnosis and treatment initiation was short. The diagnostic lost to follow-up rate was 2.1%, lower than the 5.2% reported in previous studies in Cambodia in which no mobile unit was deployed [7] and a systematic review [27]. Moreover, when CXRs were taken and interpreted on the same day the sputum was collected, same-day diagnosis more than doubled.

### Strengths and limitations

The study has several strengths. Our findings are from a massive number of screenings and diagnostic tests, and allow comparisons of different ACF approaches. Data were collected rigorously and a system was put in place to identify and clean missing and impossible values. Program implementers were part of the study team and used their experience when interpreting findings. We followed the STROBE guidelines for observational studies [28].

There are some important limitations. The division in risk groups was partially based on verbal screening. Clients might have forgotten or not felt comfortable to report TB history, HIV status, and other antecedents. Diagnosis of TB based on CXR and clinical judgement may have resulted in an overestimation of TB diagnoses. However, our study aimed at reflecting the reality of the ACF program, which targeted all forms of TB. Smear and Xpert testing were not done at point of care due to the health system organisation. In our study Xpert was used for both diagnosis (in patients with a negative CXR) and classification (patients diagnosed based on CXR were still tested with Xpert to identify BC PTB). Hence, our data cannot be used to confirm if CXR as an initial test increases the efficient use of Xpert. Finally, our findings may not be generalizable to other contexts, given that ACF strategies need to be adapted to the local context and locally available diagnostic tools [5,6]. Nevertheless, other ACF programs in high burden countries may learn from our findings, which show the potential of mobile CXR staffed with clinicians able to provide TB diagnosis.

## Conclusion

Mobile ACF teams can collect sputum, take and read a CXR, and provide a clinical consultation on the same day. On the spot interpretation of a CXR and clinical assessment increased the proportion of people diagnosed with clinical TB who started treatment the day they were tested. Using Xpert as first test increased the likelihood of diagnosing TB in the ‘high-risk group’. The majority of identified cases had clinically diagnosed PTB. To diagnose all TB forms, CXR should be part of the diagnostic algorithm. Using a combination of same-day CXR and Xpert for targeted ACF interventions can identify large numbers of people with TB. otherwise missed by passive case finding.

## Paper context

Active case finding should target all forms of tuberculosis (TB), including clinically diagnosed TB. However, most studies on active case finding focus on bacteriologically confirmed TB. Our study showed that algorithms using chest X-ray and Xpert MTB/RIF are effective in diagnosing all forms of TB.
